# Increased Actin Binding Is a Shared Molecular Consequence of Numerous SCA5 Mutations in β-III-Spectrin

**DOI:** 10.3390/cells12162100

**Published:** 2023-08-19

**Authors:** Alexandra E. Atang, Amanda R. Keller, Sarah A. Denha, Adam W. Avery

**Affiliations:** Department of Chemistry, Oakland University, Rochester, MI 48309, USA

**Keywords:** spectrin, actin, spinocerebellar ataxia, SCA5

## Abstract

Spinocerebellar ataxia type 5 (SCA5) is a neurodegenerative disease caused by mutations in the *SPTBN2* gene encoding the cytoskeletal protein β-III-spectrin. Previously, we demonstrated that a L253P missense mutation, localizing to the β-III-spectrin actin-binding domain (ABD), causes increased actin-binding affinity. Here we investigate the molecular consequences of nine additional ABD-localized, SCA5 missense mutations: V58M, K61E, T62I, K65E, F160C, D255G, T271I, Y272H, and H278R. We show that all of the mutations, similar to L253P, are positioned at or near the interface of the two calponin homology subdomains (CH1 and CH2) comprising the ABD. Using biochemical and biophysical approaches, we demonstrate that the mutant ABD proteins can attain a well-folded state. However, thermal denaturation studies show that all nine mutations are destabilizing, suggesting a structural disruption at the CH1-CH2 interface. Importantly, all nine mutations cause increased actin binding. The mutant actin-binding affinities vary greatly, and none of the nine mutations increase actin-binding affinity as much as L253P. ABD mutations causing high-affinity actin binding, with the notable exception of L253P, appear to be associated with an early age of symptom onset. Altogether, the data indicate that increased actin-binding affinity is a shared molecular consequence of numerous SCA5 mutations, which has important therapeutic implications.

## 1. Introduction

Spinocerebellar ataxia type 5 (SCA5) is a neurodegenerative disease stemming from autosomal dominant mutations in the *SPTBN2* gene encoding the cytoskeletal protein β-III-spectrin. β-III-spectrin is highly expressed in cerebellar Purkinje cells [1], the cell population targeted in SCA5 pathogenesis [2]. Within Purkinje cells, β-III-spectrin localizes to the soma and dendrites [1,3]. Mouse and cultured neuron models showed that β-III-spectrin function is required for normal dendritic arborization, spine morphology, and synaptic signaling [3,4,5,6,7]. Structurally, β-III-spectrin contains an N-terminal actin-binding domain (ABD), followed by seventeen spectrin-repeat domains, and a C-terminal pleckstrin homology domain. Analogous to other β-spectrins, the functional unit of β-III-spectrin is considered a heterotetramer composed of two β-III-spectrin subunits and two α-II-spectrin subunits. Super-resolution imaging of cortically localized β-III-spectrin supports a role for the β-III/α-II-spectrin heterotetramer in crosslinking actin filaments beneath the plasma membrane [7,8]. This spectrin-actin network, together with the spectrin adaptor protein, ankyrin-R, is likely important for maintaining proper subcellular localization of post-synaptic membrane proteins [3,9,10]. Many SCA5 mutations are known to localize to the ABD of β-III-spectrin [11], supporting the importance of actin binding and the formation of a spectrin-actin network to β-III-spectrin function.

The initial mapping of three SCA5 mutations to the *SPTBN2* gene was performed in 2006 [2]. These mutations, which included an ABD-localized L253P missense mutation, are associated with atrophy of the cerebellar cortex, and adult onset of symptoms included progressive limb and gait ataxia, slurred speech, and abnormal eye movements [12,13,14]. More recently, additional heterozygous, dominant *SPTBN2* missense mutations have been reported that are associated with early onset (infantile to adolescent) of symptoms. In addition to cerebellar atrophy and ataxia, these early onset cases are characterized by delayed motor development and sometimes intellectual disability [11,15,16,17]. These more severe symptoms resemble those of the neurodevelopmental disorder SPARCA1 [18]/SCAR14 [19], caused by homozygous recessive *SPTBN2* mutations. The SPARCA1/SCAR14 mutations are truncating mutations that are typically associated with loss-of-function. Significantly, clinically normal parents heterozygous for the SPARCA1/SCAR14 loss-of-function alleles point away from haploinsufficiency as a disease mechanism for the heterozygous SCA5 mutations. For SCA5 mutations, the underlying dominant negative molecular mechanisms responsible for variability in age of onset and symptom severity are not known. Indeed, most SCA5 mutations have not been experimentally investigated at a molecular or cellular level.

We previously characterized the ABD-localized L253P missense mutation. The native L253 residue is positioned at the interface of the two calponin-homology subdomains (CH1 and CH2) comprising the ABD. Biochemically, we showed that the L253P mutation causes a ~1000-fold increase in actin-binding affinity [20]. Through biophysical approaches, including cryo-EM, we showed that high-affinity actin binding caused by the CH2-localized L253P mutation is due to an opening of the CH1-CH2 interface [21]. This alleviates CH2 occlusion of CH1, allowing CH1 to bind actin. We further showed that L253P-induced high-affinity actin binding also requires an N-terminal helix, preceding CH1, that directly binds actin [22]. In neurons, L253P reduces dendritic localization of β-III-spectrin and induces dendritic arbor defects in cultured Purkinje cells [7] and Drosophila sensory neurons [23]. Truncation of the N-terminal helix preceding CH1 abolishes L253P-induced high-affinity actin binding and rescues L253P-induced dendritic arbor defects and neurotoxicity in Drosophila [22], supporting aberrant actin binding as a pathogenic mechanism. The way in which other ABD-localized SCA5 mutations impact the structure and function of the ABD has yet to be explored.

In the present work, we characterize the molecular consequences of nine additional, heterozygous, missense mutations localizing to the β-III-spectrin ABD. Unlike L253P, many of these mutations are associated with early onset symptoms.

## 2. Materials and Methods

### 2.1. Protein Expression and Purification

Mutations for V58M, K61E, T62I, K65E, F16C, D255G, T271I, Y272H, and H278R were introduced into the β-III-spectrin ABD using the previously generated pE-SUMO-ABD WT construct [21], through site-directed PCR mutagenesis (PfuUltra High-Fidelity DNA Polymerase, Agilent, Santa Clara, CA, USA) using the primers listed in Table 1. The resulting mutant DNAs were sequence verified. The previously generated pE-SUMO-ABD-L253P construct was utilized for subsequent experiments [22]. pE-SUMO-ABD constructs were transformed into Rosetta 2 (DE3) *E. coli* (Novagen, Armstadt, Germany). 250 mL to 1 L bacteria cultures were grown in LB broth with ampicillin (100 µg/mL) and chloramphenicol (34 µg/mL). ABD protein expression was induced with 0.5 mM IPTG, for 6 h, at 300 RPM and room temperature. Bacteria cultures were pelleted at 2987 RCF for 30 min at 4 °C, and pellets stored at −20 °C until further use. Bacterial pellets were resuspended in lysis buffer (50 mM Tris pH 7.5, 300 mM NaCl, 25% sucrose, and protease inhibitors (Complete Protease Inhibitor tablet, EDTA-free, Roche, Mannheim, Germany)), and lysed by incubation with lysozyme (Sigma, St. Louis, MO, USA) for 1 h at 4 °C, followed by a freeze–thaw cycle in an isopropanol–dry ice bath. To the lysate, MgCl_2_ (10 mM final concentration) and DNase1 (Roche, Mannheim, Germany) (8 U/mL final concentration) were added and incubated with slow stirring for 1 h at 4 °C. Lysates were clarified at 18,000 RPM at 4 °C for 30 min, in a Sorvall SS-34 rotor. Supernatants were syringe-filtered through 0.45 µm disk filters and loaded into Poly-Prep (Biorad, Hercules, CA, USA) chromatography columns containing Ni-NTA agarose (Invitrogen, Carlsbad, CA, USA) equilibrated in binding buffer containing 50 mM Tris pH 7.5, 300 mM NaCl and 20 mM imidazole. The columns were washed with binding buffer and then ABD proteins were eluted with 50 mM Tris pH 7.5, 300 mM NaCl and 150 mM imidazole. For each mutant, elution fractions containing ABD protein were pooled and loaded into a Slide-a-Lyzer, 10 K MWCO, dialysis cassette (ThermoScientific, Rockford, IL, USA), and dialysis performed overnight in 25 mM Tris pH 7.5, 150 mM NaCl, 5 mM β-mercaptoethanol, at 4 °C. 6X-His-SUMO tag was cleaved from ABD proteins using 1:10 mass ratio of Ulp1 SUMO protease:ABD in a 2 h incubation at 4 °C. Cleaved 6X-His SUMO tags and His-tagged SUMO protease were removed from the ABD proteins using 0.5–1 mL Ni-NTA agarose in a Poly-Prep chromatography column. Eluted fractions containing ABD proteins were pooled for each mutant and loaded into a Slide-a-Lyzer, 10 K MWCO, dialysis cassette for dialysis in 10 mM Tris pH 7.5, 150 mM NaCl, 2 mM MgCl_2_, 1 mM DTT, at 4 °C for 15 h. Recovered ABD proteins were measured for concentration using Bradford assay, snap-frozen in liquid nitrogen, and stored at −80 °C for future use.

### 2.2. Circular Dichroism Measurements

Purified ABD proteins were thawed and clarified at 43,000 RPM at 4 °C for 30 min in a Beckman TLA 100.3 rotor. Bradford assay (Biorad, Hercules, CA, USA) was used to determine the ABD protein concentrations. ABD proteins were diluted to 150–200 ng/µL in buffer containing 10 mM Tris pH 7.5, 150 mM NaCl, 2 mM MgCl_2_, and 1 mM DTT. CD spectra were obtained in a Jasco J-815 Spectropolarimeter with a Peltier temperature controller. A baseline correction using diluent buffer was acquired immediately before analysis. Analyses were carried out between 200 and 260 nm at 25 °C. Wavelength scans were an average of five accumulations using a 1.0 mm cuvette. Thermal denaturation studies for each ABD protein were obtained in triplicate in a Jasco J-810 or J-815 Spectropolarimeter with Peltier temperature controller. Unfolding was measured at 222 nm with increasing temperature from 20–85 °C with a pitch of 1 °C per minute. To determine the melting temperature, non-linear regression analysis was performed using Prism 8 (GraphPad Software, Inc.) using an equation for two state transition, as previously described [24], and provided here:

$$Y=(\alpha_{N}+\beta_{N}T)/\{1+e^{4T_{m}(T-T_{m})/T\Delta T}\}+(\alpha_{D}+\beta_{D}T)/\{1+e^{4T_{m}(T_{m}-T)/T\Delta T}\}$$

where *Y* is the measured signal at 222 nm, *α_N_* and *β_N_* are intercept and slope of native state, *T* is the measured temperature, ∆*T* is the unfolding transition width, *T_m_* is the fit melting temperature, and *α_D_* and *β_D_* are the intercept and slope of the denatured state. Constraints are as follows: *α_N_* > −0.2, *β_N_* and *β_D_* > 0 and *α_D_* < 1.1.

### 2.3. F-Actin Co-Sedimentation Assays

F-actin was prepared and purified from rabbit skeletal muscle (Pel-Freez Biologicals, Rogers, AR, USA), as previously described [22]. Purified ABD proteins were thawed and clarified at 43,000 RPM at 4 °C for 30 min in a Beckman TLA 100.3 rotor. Bradford assay (Biorad, Hercules, CA, USA) was used to determine the F-actin and ABD protein concentrations. As performed previously [22], binding reactions were prepared with 2 µM ABD protein and increasing concentrations of F-actin (3–120 µM) in F-buffer (10 mM Tris pH 7.5, 150 mM NaCl, 0.5 mM ATP, 2 mM MgCl_2_, and 1 mM DTT). After 30 min incubation at room temperature, binding reactions were centrifuged at 50,000 RPM for 30 min at 25 °C in a TLA-100 rotor (Beckman, Brea CA, USA). Promptly after centrifugation, supernatants were sampled and mixed with Laemmle sample buffer (Biorad, Hercules, CA, USA). Collected supernatant samples were separated by SDS-PAGE and bands were visualized using Coomassie Brilliant Blue R-250 (Biorad, Hercules, CA, USA) solution. After sufficient destaining to remove background, gels were imaged using the 680 nm channel of an Azure Sapphire imager. ABD protein band fluorescence intensities were quantified from image files using Image Studio Lite version 5.2 software (LI-COR Biosciences, Lincoln, NE, USA). A standard curve was generated using various amounts of ABD proteins on a Coomassie stained gel to relate ABD fluorescence intensity to known ABD concentrations. The standard curves were used to convert raw fluorescence ABD signals to concentration for the binding assays. Using Prism 8 (GraphPad Software, Inc., Boston, MA, USA), dissociation constants (*Kd*) were determined through a nonlinear regression fit for a one-site specific binding equation, *Y* = *X*/(*Kd* + *X*), where *Y* is the bound ABD fraction and *X* is the free F-actin concentration [25], with Bmax constrained to 1, as described previously [20].

### 2.4. Structural Modeling

The β-III-spectrin ABD structural homology model was generated through the i-Tasser server [26]. The top template structure was the plectin ABD (PDB ID: 1MB8). The crystal structure of β-II-spectrin calponin homology domain 2 (PDB ID: 1BKR) was used for alignment with the generated β-III-spectrin ABD homology model. Analyses of the structures, including identification of predicted contacts for different mutated residues, and alignment, was performed with PyMOL v2.5.4 (Schrödinger, New York, NY, USA).

### 2.5. Statistical Analysis

Statistical significance was determined in Prism 8 (GraphPad Software, Inc., Boston, MA, USA) for all melting temperatures and dissociation constants (above 1 µM) by one-way ANOVA, followed by Dunnett multiple comparisons post hoc test. *p*-values less than 0.05 were deemed significant.

## 3. Results

### 3.1. Clinical Summary of ABD-Localized Mutations

Here we report the molecular characterization of nine ataxia-associated, ABD-localized, missense mutations in β-III-spectrin. These mutations include V58M, K61E, T62I, K65E, F160C, D255G, T271I, Y272H, and H278R. All mutations were heterozygous in patients, consistent with a dominant mechanism of action. T62I, K65E, F160C, D255G, and T271I were identified in patients with infantile onset (<12 mo) symptoms [11,15,16,27]. The compound heterozygous mutation Y272H/W2065* was identified in an infantile-onset patient [16]. Notably, parents carrying a single mutated allele (Y272H/+ or W2065*/+) were clinically normal (age of parents not reported). Thus, Y272H appears to genetically interact with W2065* to cause disease. This suggests that Y272H by itself is either insufficiently disruptive to cause symptoms, or the mutation causes mild, late onset symptoms not yet presenting in the Y272H/+ parent. For this study, only the normal clinical phenotype of the heterozygous (Y272H/+) parent will be considered. H278R was associated with adolescent symptom onset (11 years) [28]. K61E was early onset at an unconfirmed age but was evaluated at 9 years [27]. V58M was associated with adult onset (31 years) [27]. In addition to T271I and T62I characterized in this study, T271N [29] and T62N [30] mutations, not characterized here, are also associated with early age of onset. Additionally, another recently reported ABD-localized mutation, I162M [31], is associated with late onset of symptoms, but is not characterized in this study. A detailed summary of clinical features is provided in Table 2.

### 3.2. ABD-Localized Mutations Cluster to the CH1-CH2 Interface

**Figure 1:**
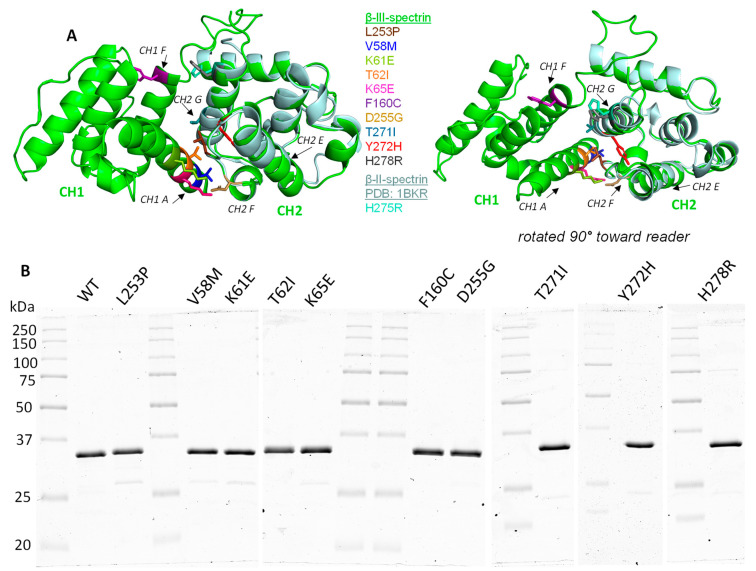
Position of SCA5 missense mutations in the actin-binding domain, and purification of mutant ABDs. (**A**) Homology model of β-III-spectrin actin-binding domain (ABD; green), containing CH1 and CH2 subdomains (**left**) and a second view rotated 90° toward the reader (**right**). Amino acids are color-coded for sites of mutations. The crystal structure of the isolated β-II-spectrin CH2 domain (blue–silver) is aligned with β-III-spectrin CH2. All mutated residues localize to the interface of the CH1 and CH2 subdomains. (**B**) Coomassie blue stained gel images showing purified wild-type (WT) and mutant ABD proteins. All ABD proteins run at the predicted size of 32 kDa.

To explore the position of the mutations in the β-III-spectrin ABD, a structural homology model was generated using iTasser [26], in which the plectin ABD (PDB ID: 1MB8) was the top template. The β-III-spectrin ABD is comprised of two subdomains—calponin homology domains 1 and 2 (CH1 and CH2), Figure 1A. Strikingly, all of the mutations are positioned at or near the interface of CH1 and CH2, such as L253P. The native V58, K61, T62, and K65 amino acids localize to CH1 helix A and are predicted to directly contact CH2 residues. Notably, T62 and K65 directly contact CH2 residue L253, interactions we previously highlighted in our characterization of the L253P mutation [20]. The native F160 residue localizes to CH1 helix F. The F160 sidechain is not directly oriented towards CH2. However, F160 is predicted to contact CH1 residue, W66, which in turn contacts CH2 residues L253 and T271. Mutations localizing to CH2 include D255G, T271I, Y272H, H278R, and L253P. The native D255 localizes to a loop connecting CH2 helices E and F and is predicted to form salt bridges with CH1 residues K61 and K65. T271, Y272, and H278 localize to CH2 helix G. T271 is predicted to directly contact CH1 residues W66 and I157, in addition to contacting CH2 residue L253. Y272 forms extensive hydrophobic contacts with L253, suggesting that Y272 plays an important role in coordinating the position of L253. In the homology model, H278, while oriented towards CH1, is not predicted to contact CH1 residues. To further evaluate the position of H278, we analyzed the equivalent amino acid (H275) in a crystal structure of the isolated CH2 subdomain of β-II-spectrin [32]. Alignment of the β-II-spectrin CH2 domain with the β-III-spectrin ABD homology model shows that β-II-spectrin H275 is oriented towards CH1 and predicted to contact CH1 Q161. Thus, this suggests that β-III-spectrin H278 also contacts CH1 Q161. In summary, the mutated residues cluster to the CH1-CH2 interface. Many of the mutated residues contact each other, forming an interaction network that is likely important for bridging CH1 and CH2. The common position of these mutations at the CH1-CH2 interface suggests that the mutations have similar structural and functional impacts on the ABD.

### 3.3. SCA5 Mutant ABDs Can Attain a Well-Folded State, but Are Destabilized

**Figure 2:**
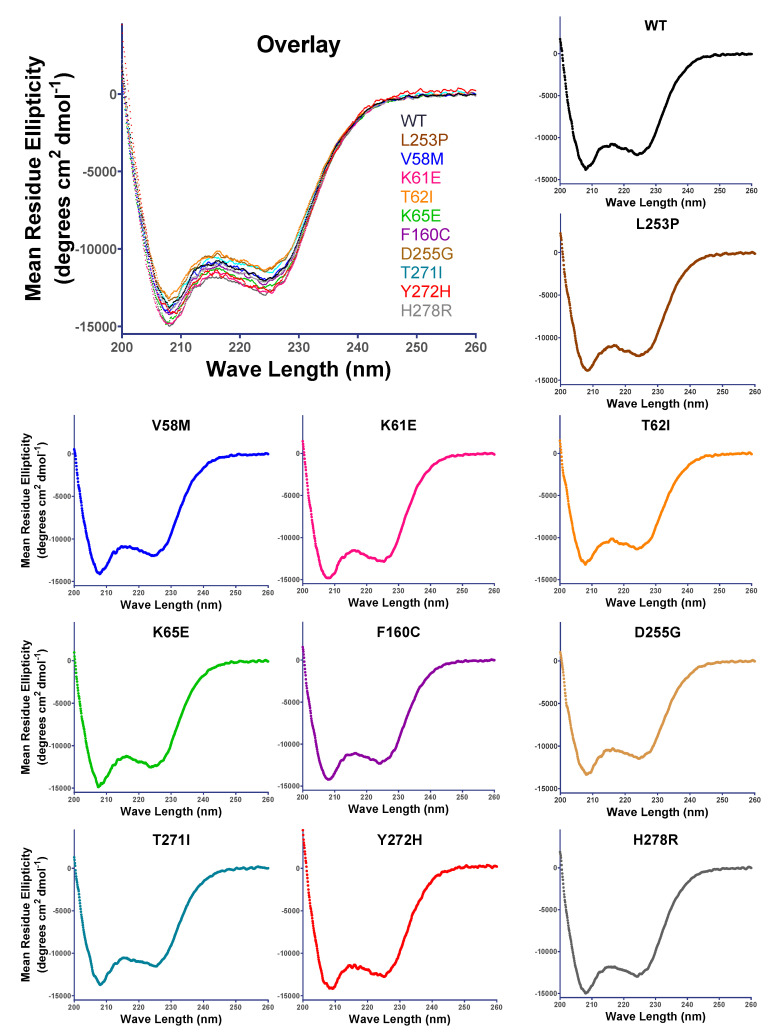
Circular dichroism absorption spectra show α-helical profiles for wild-type and mutant ABDs. CD spectra between 200 nm and 260 nm for individual wild-type, L253P, V58M, K61E, T62I, K65E, F160C, D255G, T271I, Y272H, and H278R ABD proteins. Minima at 208 and 222 nm are characteristic of α-helical fold. Spectra are average of n = 5 accumulations.

**Figure 3:**
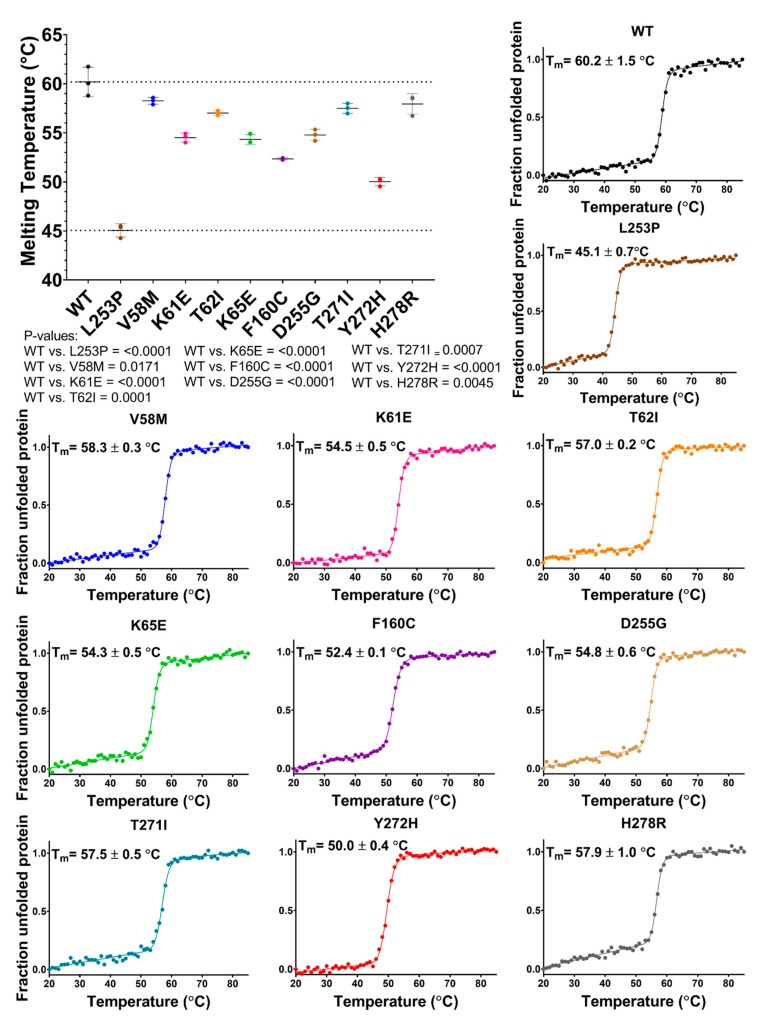
Thermal denaturation curves showing that all ABD proteins unfold cooperatively, but mutants are destabilized. Melting curves of purified ABD proteins, generated by monitoring circular dichroism absorption at 222 nm while heating the sample. The cooperative, two-state, transitions indicate well-folded proteins. All mutant ABD proteins have Tm’s that are significantly lower than wild-type. Statistical significance was determined by one-way ANOVA, followed by multiple comparisons post hoc test. *p*-values are reported below the upper left panel. Tm = average +/− standard deviation, with n = 3.

To test how the SCA5 mutations impact the structure and function of the ABD, we expressed the wild-type and mutant ABD proteins in *E. coli* and performed protein purification. All ABD proteins were purified to homogeneity or contained only minor contaminating bands, Figure 1B. To determine if the SCA5 mutations impact the folded state of the ABD, circular dichroism (CD) spectroscopy was performed. Similar to our prior characterization [20], the wild-type ABD shows an α-helical absorption profile with characteristic minima at 208 and 222 nm, Figure 2. Similarly, all ABD mutants showed pronounced α-helical profiles. From this, we conclude that all the mutant ABDs can attain a well-folded state without significant disruption to secondary structure. To determine how the mutations impact the stability of the ABD, CD thermal denaturation studies were performed. The wild-type ABD unfolded in a cooperative manner, characterized by a two-state transition and a melting temperature (Tm) of 60.2 ± 1.5 °C (Figure 3), consistent with our prior report [20]. All mutant ABD proteins also displayed cooperative unfolding, further supporting a well-folded state for the mutant proteins. However, the mutants unfolded at statistically significant lower temperatures relative to wild-type, with Tm values ranging from 50.0 ± 0.4 to 58.3 ± 0.3 °C. An exception was L253P, which unfolded with a much lower Tm, 45.1 ± 0.7 °C, consistent with our prior characterization [20]. We previously showed that L253P protein destabilization is associated with an opening of the CH1-CH2 interface [21]. Thus, the current denaturation studies, together with the common position of the mutations at the CH1-CH2 interface, strongly suggest that all the mutations have a shared effect to structurally destabilize the CH1-CH2 interface.

### 3.4. All of the SCA5 Mutations Increase Actin-Binding Affinity

**Figure 4:**
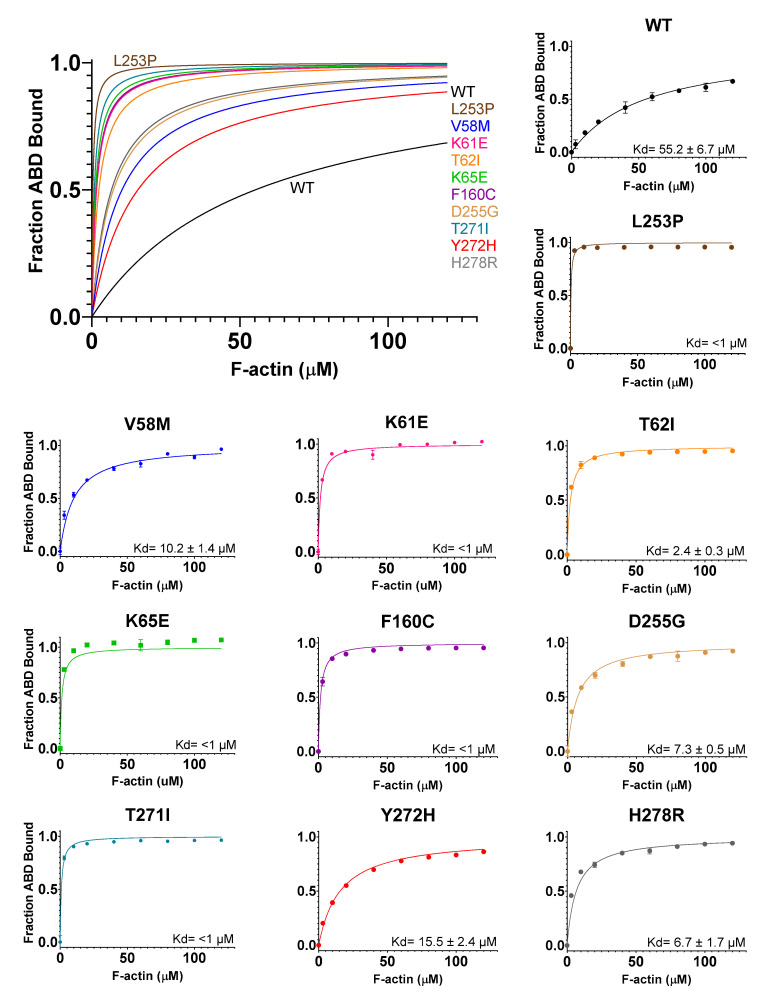
Actin co-sedimentation assays show that all mutations increase actin-binding affinity. Co-sedimentation of ABD proteins and actin reveal greater actin affinities for all mutant ABD proteins compared to wild-type. Representative binding data and curve fit is shown for each mutant ABD protein. Kd = average +/− standard deviation, with n = 6 or more binding assays.

Previously, we showed that the L253P mutation causes a ~1000-fold increase in actin-binding affinity, with an estimated Kd of 75 nM [20]. To determine how the additional SCA5 mutations impact actin binding, in vitro co-sedimentations assays were performed using the purified ABD proteins. In these binding assays, a fixed amount of ABD (2 µM) was mixed with increasing concentrations of F-actin (3–120 µM), and the amount of ABD bound to actin filaments was determined after pelleting actin in an ultracentrifuge. The wild-type ABD bound F-actin with a Kd of 55.2 ± 6.7 µM, Figure 4. Significantly, all of the mutant ABDs bound actin with higher affinity than wild-type. Consistent with an estimated 75 nM Kd, the L253P ABD was entirely bound to F-actin at all F-actin concentrations tested (3 µM to 120 µM). With the exception of the lowest actin concentrations (3–10 µM), the K61E, K65E, F160C, and T271I mutant ABDs were also fully bound to actin. This indicates that K61E, K65E, F160C, and T271I mutations also cause high-affinity actin binding (submicromolar Kd). In contrast, V58M, T62I, D255G, Y272H, and H278R mutations caused more modest increases in actin affinity. The Kd values for V58M, T62I, D255G, Y272H, and H278R were statistically significant relative to wild-type, and equaled 10.2 ± 1.4 µM, 2.4 ± 0.3 µM, 7.3 ± 0.5 µM, 15.5 ± 2.4 µM, and 6.7 ± 1.7 µM, respectively. In sum, all of the mutations cause increased actin-binding affinity, consistent with the shared position of these mutations at the CH1-CH2 interface. Notably, the actin-binding affinities vary greatly among the different mutations, with L253P causing the highest affinity and Y272H causing the smallest increase in affinity.

## 4. Discussion

Here we build upon our prior studies of the L253P mutation by characterizing nine additional, ABD-localized missense mutations. Strikingly, our structural analyses revealed that all of the newly characterized mutations, such as L253P, localize to the CH1-CH2 interface. Many of the native residues are predicted to contact each other, forming an interaction network that is likely important for bridging CH1 and CH2. Our circular dichroism analyses showed that the mutations are destabilizing, likely reflecting a structural uncoupling of CH1 and CH2, as we showed previously for L253P [21]. Our prior studies of the L253P mutation [21], along with studies of spectrin-related ABD proteins [33,34,35], established that opening the CH1-CH2 interface is required for binding CH1 to actin. Consistent with the position of the newly characterized mutations at the CH1-CH2 interface, and the destabilizing impact of these mutations, we showed that all of the mutations cause increased actin binding. Thus, SCA5 mutations localizing to the ABD appear to act through a common molecular mechanism. It will be important to further validate a shared disease mechanism using in vivo approaches, for example, with mouse models, cultured Purkinje neurons, or patient-based materials.

Our prior studies of the L253P mutation in Drosophila suggest that high-affinity actin binding mislocalizes the spectrin cytoskeleton in neurons, causing the mutant β-spectrin and associated proteins to accumulate in the soma and be depleted in the dendritic arbor [23]. Consequently, distal dendrites degenerate, and dendritic arbor outgrowth is reduced. Recently, a similar mislocalization of L253P β-III-spectrin was reported in transfected mouse Purkinje neurons [7]. Here, the L253P mutant caused misorientation of dendrites, resulting in reduced planarity of the Purkinje cell dendritic arbor. If increased actin binding drives pathogenesis for all ABD-localized SCA5 mutations, then we expect that the nine newly characterized mutations will result in spectrin mislocalization and arborization defects in these neuronal systems, similar to L253P. However, for the newly characterized mutations, the observed neuronal defects may be less severe due to the lesser effect of these mutations to increase actin-binding affinity, relative to L253P.

A potentially important finding from the present study is that the actin-binding affinities of the mutants vary greatly. Perhaps this variability in actin-binding affinity contributes to the variability in age of onset (and symptom severity). In Table 3, our experimentally determined Kd values are listed for each of the mutations, along with clinical data on age of symptom onset. As evident in Table 3, most of the nine newly characterized mutations are associated with early onset of symptoms (infant to adolescent). These early onset mutations are associated with single digit micromolar to submicromolar Kd values. In contrast, with a Kd value of 10 µM, V58M is associated with adult onset of symptoms (37 y). Furthermore, Y272H, with a Kd of 16 µM, causes the smallest increase in actin binding among the mutations. A Y272H/+ parent, of unknown age, was reported to be clinically normal [16]. This supports that a modest increase in actin binding can be tolerated (no clinical phenotype), and that symptoms are more likely to present as actin-binding affinity increases. In summary, a correlation appears to be emerging in which high-affinity actin binding (submicromolar to single digit micromolar Kd) is associated with early age of symptom onset, and that smaller increases in actin-binding affinities (10 micromolar or higher Kd) are associated with later onset or no disease symptoms. However, a strong correlation is still difficult to establish, as most of the mutations are de novo, and n values equal 1. Thus, it is important to collect clinical data on age of onset from additional patients carrying the same mutations, and to further characterize distinct ABD-localized, ataxia-associated mutations, as they are reported. For example, the recently reported T62N and T271N mutations are early onset and would be expected to have submicromolar to single digit micromolar Kd values. In contrast, a CH1-CH2 interface mutation, I162M, causes late onset symptoms, and would be expected to have a higher Kd value. Importantly, the L253P mutation, which causes the highest actin-binding affinity but is associated with late onset of symptoms (mean = 32.8 y), is a major outlier to our proposed association between actin-binding affinity and age of onset, as discussed below.

Our data show that L253P is unique among the mutations in that it causes (1) the highest actin-binding affinity and (2) the greatest protein destabilization. It thus seems likely that the decreased clinical severity of L253P is attributed to one or both of these features. Our current and prior [20] thermal denaturation studies show in vitro that L253P ABD begins to unfold near physiological temperature. It is thus possible that L253P β-III-spectrin is prone to denaturation and enhanced protein turnover in cells at physiological temperature. Increased protein turnover of the L253P mutant would reduce its abundance in neurons, corresponding to a reduction in neurotoxicity and a later age of symptom onset. However, in transiently transfected HEK293T cells, wild-type and L253P β-III-spectrin ABD or full-length proteins showed similar protein levels by western blot analyses [23]. Clarkson et al. additionally showed that L253P β-III-spectrin did not induce pathways associated with unfolded protein response [36]. Moreover, we showed that L253P, and a L253A mutation, which causes high-affinity actin binding but is less destabilizing than L253P, cause similar mislocalization (absence from plasma membrane extensions and internal accumulation on actin-rich vesicles) of β-III-spectrin in HEK293T cells [23]. This supports that the behavior of the L253P mutant β-III-spectrin in cells is driven by elevated actin binding, not protein denaturation/unfolding. Alternatively, it is possible that high-affinity actin binding localizes the L253P mutant to a distinct population of actin filaments not bound by the ABD mutants with relatively lower actin-binding affinity. Future studies assessing the impact of L253P versus the other ABD mutations on mutant β-III-spectrin steady-state protein levels and subcellular localization in cultured Purkinje neurons, mouse models, or patient-based materials may clarify why L253P has reduced toxicity/late onset symptoms.

Our finding here that increased actin-binding affinity is a shared molecular consequence of numerous SCA5 mutations has important therapeutic implications. Specifically, it suggests that a small molecule drug that can alleviate high-affinity actin binding may be broadly useful as a SCA5 therapeutic. Recently, we reported the identification of several small molecules capable of reducing binding of the L253P mutant to actin in vitro [37]. This established that the spectrin-actin interaction can be targeted by small molecules. These compounds will potentially be effective in reducing actin binding caused by the multiple different SCA5 mutations characterized here. Our analysis of Kd versus age of symptom onset suggests that reducing actin-binding affinity to a Kd value of ~16 µM (Y272H) or higher may be needed to fully eliminate toxicity caused by the ABD mutations.

## Figures and Tables

**Table 1:** Sequence of DNA primers used in this study. The following primers were used to introduce SCA5 mutations into the β-III-spectrin ABD coding sequence and are listed 5′ to 3′.

Mutation	Forward Primer	Reverse Primer
V58M	CTGGCAGATGAACGAGAAGCTATGCAGAAGAAAACCTTCACCAAG	CTTGGTGAAGGTTTTCTTCTGCATAGCTTCTCGTTCATCTGCCAG
K61E	GAACGAGAAGCTGTGCAGAAGGAAACCTTCACCAAGTGGGTAAAC	GTTTACCCACTTGGTGAAGGTTTCCTTCTGCACAGCTTCTCGTTC
T62I	GAGAAGCTGTGCAGAAGAAAATCTTCACCAAGTGGGTAAACTC	GAGTTTACCCACTTGGTGAAGATTTTCTTCTGCACAGCTTCTC
K65E	GTGCAGAAGAAAACCTTCACCGAGTGGGTAAACTCGCACCTGGCC	GGCCAGGTGCGAGTTTACCCACTCGGTGAAGGTTTTCTTCTGCAC
F160C	GTCTGGACCATCATCCTTCGATGCCAGATCCAAGACATCAGTGTG	CACACTGATGTCTTGGATCTGGCATCGAAGGATGATGGTCCAGAC
D255G	GGACTTACCAAGCTGCTGGGTCCCGAAGACGTGAATGTGGACC	GGTCCACATTCACGTCTTCGGGACCCAGCAGCTTGGTAAGTCC
T271I	GCCAGATGAGAAGTCAATCATTATCTATGTGGCTACTTACTACC	GGTAGTAAGTAGCCACATAGATAATGATTGACTTCTCATCTGGC
Y272H	GATGAGAAGTCAATCATTACCCATGTGGCTACTTACTACCATTAC	GTAATGGTAGTAAGTAGCCACATGGGTAATGATTGACTTCTCATC
H278R	CTATGTGGCTACTTACTACCGTTACTTCTCCAAGATGAAG	CTTCATCTTGGAGAAGTAACGGTAGTAAGTAGCCACATAG

**Table 2:** Clinical presentations of ataxia-associated mutations in the actin-binding domain of β-III-spectrin. Mutations not characterized in this study are shaded in grey.

*SPTBN2* Gene Mutation	Inheritance	Clinical Onset, Sex	Age at Last Evaluation	Clinical Features	Brain MRI Findings
c. 172 G > A (p. V58M) [27]	familial	37 y, M n = 1	61 y	Progressive cerebellar ataxia; Memory problems	Cerebellar Atrophy (Mild, stable)
heterozygous					
c. 181 A > G (p. K61E) [27]	de novo	Unknown, F n = 1	9 y	Cerebellar ataxia; Hyperreflexia; Hypotonia; Dysarthria; Nystagmus	Cerebellar Atrophy
heterozygous					
c.185 C >T (p. T62I) [16]	not known	8 mo, F n = 1	18 y	Cerebellar ataxia; Cognitive delay; Psychomotor delay; Microcephaly; Nystagmus; Hypotonia; Mild bradykinesia	Cerebellar Atrophy
heterozygous					
c. 185 C > A (p. T62N) [30]	de novo	5 mo, M n = 1	1 y 4 mo	Cerebellar ataxia; Hypotonia; Brisk reflexes; Motor and cognitive developmental delay; Nystagmus; Strabismus	Severe Cerebellar Atrophy
heterozygous					
c. 193 A > G (p. K65E) [11]	de novo	4 mo, M n = 1	11 y	Ataxia; Hypotonia; Ataxic gait; Dysarthria (understandable); Strabismus; Horizontal nystagmus; Dysmetria; Intention tremor (mild)	Progressive Cerebellar Atrophy
heterozygous					
c.479 C > T (p. F160C) [16]	de novo	5 mo, M n = 1	5 y	Cerebellar ataxia; Psychomotor delay; Strabismus; Hypotonia; Cognitive delay; Unable to walk; Nonverbal	Progressive Cerebellar Atrophy
heterozygous					
c. 486 C > G (p. I162M) [31]	familial	48 y, F n = 1	53 y	Cerebellar ataxia; Dysarthria; Ataxic gait	Moderate Cerebellar Atrophy
heterozygous					
c. 758 T > C (p. L253P) [12]	familial	15–50 y, mean = 32.8 y, n = 15	20–62 y	Stance, gait, and limb ataxia (14/15); Dysarthria (13/15); Intention tremor (5/15); Rest tremor (2/15); Nystagmus (13/15)	Cerebellar Atrophy
heterozygous					
c. 764 A > G (p. D255G) [11]	de novo	1 y, F n = 1	8 y	Ataxia; Strabismus; Hypotonia; Bradykinesia; Ataxic gait; Dysarthria (difficult to understand); Intention tremor (moderate); ADHD	Progressive Cerebellar Atrophy
heterozygous					
c.812 C > T (p. T271I) [15]	de novo	6 mo, M n = 1	8 y	Cerebellar ataxia; Motor developmental delay; Brisk reflexes; Ataxic gait and limb movements; Dystonia; Intention tremor	Progressive Cerebellar Atrophy
heterozygous					
c. 812 C > A (p. T271N) [29]	de novo	Unknown n = 1	4 y 7 mo	Ataxia; Psychomotor retardation	Cerebellar Hypoplasia
heterozygous					
c.888 T > C; + (p. Y272H/+) [16]	familial	Unknown n = 1	Adulthood	No symptoms	NA
heterozygous					
c.833 A > G (p. H278R) [28]	de novo	11 y, F n = 1	19 y	Cerebellar ataxia; Ataxic gait; Nystagmus	Cerebellar Atrophy

**Table 3:** Actin-binding affinity and age of symptom onset for different ABD mutations.

Mutation	Kd (µM)	Age of Onset
L253P	<1	15–50 y, mean = 32.8 y, n = 15
T271I	<1	6 mo, n = 1
K65E	<1	4 mo, n = 1
F160C	<1	5 mo, n = 1
K61E	<1	Not reported; clinical evaluation at 9 y, n = 1
T62I	2.4 ± 0.3	8 mo, n = 1
H278R	6.7 ± 1.7	11 y, n = 1
D255G	7.3 ± 0.5	12 mo, n = 1
V58M	10.2 ± 1.4	37 y, n = 1
Y272H/+	15.3 ± 2.0	Y272H/+ parent is clinically normal, n = 1
Wild-type	55.2 ± 6.7	NA

## Data Availability

All data are contained within the manuscript.
